# A 33,000-Year-Old Incipient Dog from the Altai Mountains of Siberia: Evidence of the Earliest Domestication Disrupted by the Last Glacial Maximum

**DOI:** 10.1371/journal.pone.0022821

**Published:** 2011-07-28

**Authors:** Nikolai D. Ovodov, Susan J. Crockford, Yaroslav V. Kuzmin, Thomas F. G. Higham, Gregory W. L. Hodgins, Johannes van der Plicht

**Affiliations:** 1 Siberian Branch of the Russian Academy of Sciences, Institute of Archaeology and Ethnography, Novosibirsk, Russia; 2 Pacific Identifications Inc., Victoria, British Columbia, Canada; 3 Siberian Branch of the Russian Academy of Sciences, Institute of Geology and Mineralogy, Novosibirsk, Russia; 4 Research Laboratory for Archaeology and the History of Art, Oxford University, Oxford, United Kingdom; 5 National Science Foundation-Arizona Accelerator Mass Spectrometry Laboratory, University of Arizona, Tucson, Arizona, United States of America; 6 Center for Isotope Research, Groningen University, Groningen, The Netherlands; 7 Faculty of Archaeology, Leiden University, Leiden, The Netherlands; Paleontological Institute of Russian Academy of Science, United States of America

## Abstract

**Background:**

Virtually all well-documented remains of early domestic dog (*Canis familiaris*) come from the late Glacial and early Holocene periods (ca. 14,000–9000 calendar years ago, cal BP), with few putative dogs found prior to the Last Glacial Maximum (LGM, ca. 26,500–19,000 cal BP). The dearth of pre-LGM dog-like canids and incomplete state of their preservation has until now prevented an understanding of the morphological features of transitional forms between wild wolves and domesticated dogs in temporal perspective.

**Methodology/Principal Finding:**

We describe the well-preserved remains of a dog-like canid from the Razboinichya Cave (Altai Mountains of southern Siberia). Because of the extraordinary preservation of the material, including skull, mandibles (both sides) and teeth, it was possible to conduct a complete morphological description and comparison with representative examples of pre-LGM wild wolves, modern wolves, prehistoric domesticated dogs, and early dog-like canids, using morphological criteria to distinguish between wolves and dogs. It was found that the Razboinichya Cave individual is most similar to fully domesticated dogs from Greenland (about 1000 years old), and unlike ancient and modern wolves, and putative dogs from Eliseevichi I site in central Russia. Direct AMS radiocarbon dating of the skull and mandible of the Razboinichya canid conducted in three independent laboratories resulted in highly compatible ages, with average value of ca. 33,000 cal BP.

**Conclusions/Significance:**

The Razboinichya Cave specimen appears to be an incipient dog that did not give rise to late Glacial – early Holocene lineages and probably represents wolf domestication disrupted by the climatic and cultural changes associated with the LGM. The two earliest incipient dogs from Western Europe (Goyet, Belguim) and Siberia (Razboinichya), separated by thousands of kilometers, show that dog domestication was multiregional, and thus had no single place of origin (as some DNA data have suggested) and subsequent spread.

## Introduction

The dog is the oldest domesticated animal, and patterns of its earliest occurrence are of great importance in current zoology, anthropology, and archaeology [1], [2]. Although the presence of domesticated dogs is established for about the last 14,000 calendar years (cal BP) [3], [4], the existence of dogs prior to the Last Glacial Maximum (LGM), ca. 26,500–19,000 cal BP [5], is unresolved [2]. A dog-like canid skull, recently reported from the Upper Paleolithic site of Goyet (Belgium) (50°24′N, 05°04′E) with a direct age of ca. 36,000 cal BP [6], raises questions about the time and place of the earliest domestication of the dog. The large size of the Goyet skull and other very early canid material [6], [7] hampers the determination of whether these earliest remains represent domesticated dogs rather than wolves with a few cranial features typical of dogs.

Morphological characteristics remain the most reliable criterion for separation of domesticated dogs from wolves [1]. The results of DNA analyses of modern dogs and wolves are contradictory, with China [8], [9] and the Middle East [10] suggested as the exclusive setting for initial domestication of dog. Direct age determination of putative early dogs (rather than assumed dates of associated other bones or charcoal) is crucial and Accelerator Mass Spectrometry (AMS) radiocarbon dating is the best available method for establishing the antiquity of early dogs [3], [4], [6].

## Methods

### The Razboinichya Cave

**Figure 1:**
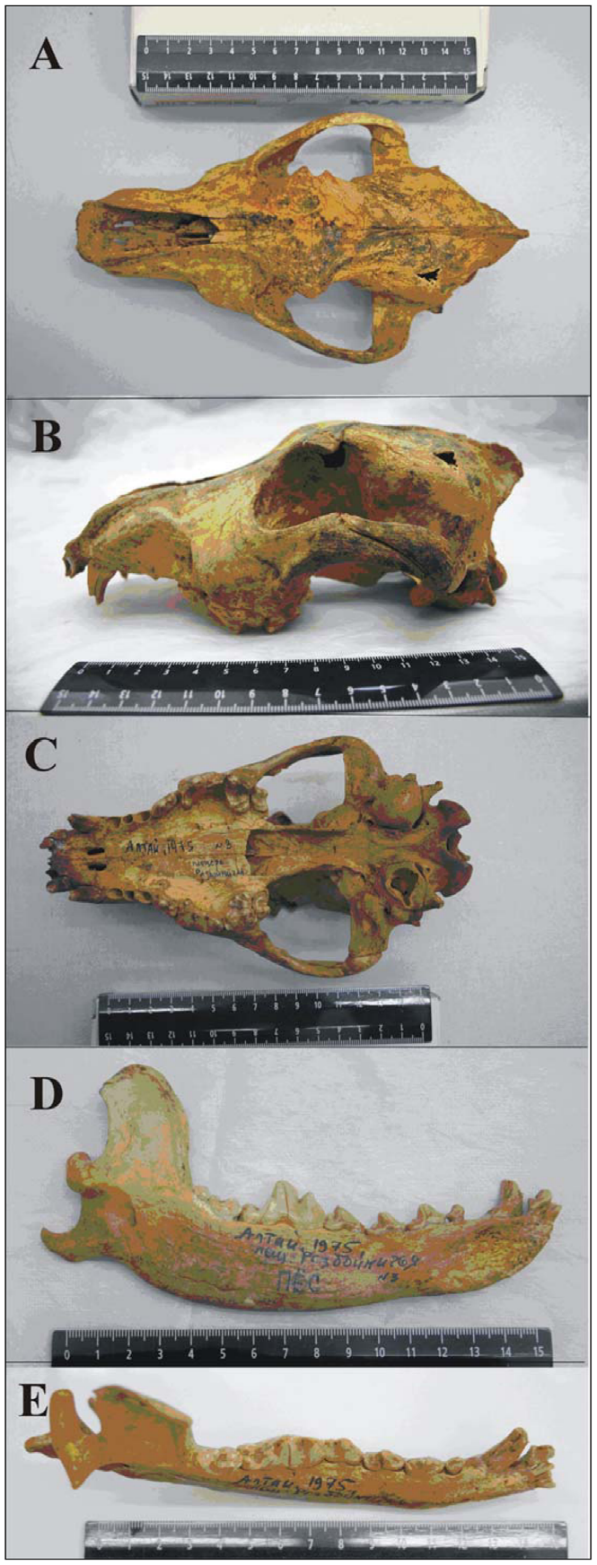
The Razboinichya canid. A) aerial view, B) profile, C) palate, D) left mandible, E) left lower tooth row (scale on ruler in cm). Sub-triangular hole in the skull is the place of initial sampling for ^14^C dating in 2007.

We were able to retrieve the complete skull and mandibles of a dog-like canid from the pre-LGM context of Razboinichya [*Bandit's*] Cave in Altai Mountains of southern Siberia (51°18′N, 84°28′E) (see Figure S1). The cave is situated in the northwestern part of Altai Mountains, in a small limestone massif, and was discovered in 1962. First paleontological survey was conducted by N. D. Ovodov in 1975, when the skull and both mandibles of a dog-like canid (Figure 1) were found, and excavations continued in 1977–1991.

The cave is 90 m long; it is almost horizontal near the entrance and up to 15 m inside; further inside, it goes down to a depth of about 19 m where there is a main chamber 3 m high and 10 m wide. In this chamber, below loose brownish-grayish loam 10–15 cm thick, the main fossil-bearing sediments (reddish-brownish loam, thickness of 40–50 cm) occur. They contain remains of permafrost and frozen mummified animal tissues, and numerous bones of mammals and birds, small pieces of charcoal, wood, and bark, as well as mollusk shells and some bones of reptiles, amphibians, and fish. Below brownish-grayish loam, there is bedrock (limestone).

In total, about 71,290 mammalian bones and bone fragments were excavated. Large mammals are represented mainly by foxes *Vulpes vulpes* and *V. corsac* (ca. 11,800 bones), cave hyena *Crocuta spelaea* (ca. 8870 bones), grey wolf *Canis lupus* (ca. 1670 bones), and brown bear *Ursus arctos* (ca. 670 bones). It seems that the chamber was primarily used in antiquity by hyenas as a den: the remains of at least 137 individual hyenas were counted and about 130 hyena coprolites were found. Brown bears and wolves also appear to have used the grotto as a den on occasion. The dog-like canid skull reported here was found in this reddish-brownish loam. Among other animals, the most numerous are ibex/sheep (*Ovis* sp./*Capra* sp.) (ca. 3900 bones) and hares (*Lepus* sp.) (ca. 1500 bones). No artifacts were recovered from this layer but the presence of small charcoal pieces and burnt bones probably testify that ancient people visited cave at least occasionally.

A single bone of brown bear from the fossil-bearing layer was radiocarbon dated to 14,850±700 BP (SOAN-1468), and this ^14^C value was initially associated with the Razboinichya dog-like canid (e.g. [11]). Several Accelerator Mass Spectrometry (AMS) ^14^C dates were later obtained on grey wolf teeth from the same layer: 32,500+270/−260 BP (KIA-25291), 48,020+1840/−1500 BP (KIA-25303), and more than 49,930 BP (KIA-25304) [12, Supplement].

### Archaeozoological study

The skull and left mandible of the Razboinichya canid were measured (see Tables S1, S2) according to standard osteological criteria [13]. The skull and both mandibles were also photographed with cm scale from several perspectives (Figure 1). We assessed the taxonomic status of the specimens using the photos and measurements provided by comparing these to data collected on canid specimens reported in published literature. It was determined essential to include European late Pleistocene wolves in this comparison, as they must be considered a likely ancestral population for early dogs in northern Eurasia. Prehistoric Greenland dogs (dated ca. 1000 BP or later) were used to represent fully domestic dogs, as they represent a large-sized but unimproved type. Specifically, the comparative samples included large Pleistocene wolves from Předmosti, Czech Republic [14] dated ca. 31,000 cal BP, modern wolves from Europe and North America [15], [16], and prehistoric Greenland dogs from the Thule period [17]. We relied on Musil's [14] informed interpretation and English translation of Pokorný's [18] original Czech-language report on Předmosti wolves, especially in determining that Pokorný's [18] measurement “P” (longest snout length) rather than “Q” (shortest snout length) was equivalent to the snout length dimension used in this analysis (#12 as defined by von den Driesch [13]), as Pokorný [18] did not provide a diagram. Actual measurements and two index ratios commonly used in such taxonomic studies (snout width and tooth crowding) were used for comparison [19], [20]. Selected mandible measurements are presented (see Table S2). Ratio diagrams were used to compare the basic skull shape of the Razboinichya canid to Pleistocene wolves, modern wolves and prehistoric dogs of similar size, as these diagrams are considered biologically informative and thus provide straightforward comparisons of size and shape [21].

### Radiocarbon dating

Sampling of the Razboinichya canid was initially conducted in 2007 on skull (Figure S2). In 2008, three sub-samples were additionally taken from the mandible (Figure S3), and dated independently at three laboratories located at Tucson, Arizona (USA), Oxford (UK), and Groningen (the Netherlands).

In Tucson (NSF-Arizona AMS Laboratory; Lab Code AA), the bone sample was cleaned with distilled water and dried. Bone was afterwards ground using a porcelain pestle and mortar, and then was demineralized at room temperature with 0.25 N HCl for 24 hours [22]. The sample was then rinsed with distilled water and gelatinized in 0.01 N HCl at 60°C for 48 hours. The gelatin was filtered through quartz paper; the filtrate lyophilized, and the recovered collagen collected and weighed. Carbon was extracted from the collagen by combustion under vacuum tubes in the presence of copper oxide (CuO). The resulting carbon dioxide gas (CO_2_) was cryogenically isolated from the other combustion gases. The CO_2_ was converted to graphite by reduction with zinc (Zn) using iron (Fe) powder as a catalyst [23]. The graphite was pressed into a target holder, which fits the carrousel of the AMS ion source. Subsequently, the ^14^C measurement was performed.

In Oxford Radiocarbon AMS Unit (ORAU; Lab Code OxA), bone powder was loaded into a glass test tube and a sequence of 0.5 M HCl, 0.1 M NaOH and 0.5 M HCl used to treat the bone, interspersed with rinsing with ultra-pure water between each reagent. Collagen was gelatinized in a pH 3 solution at 75°C for 20 hours and the gelatin filtered using a 9 µm polyethylene filter. This gelatin was then pipetted into a pre-cleaned ultra-filter and centrifuged at 2500–3000 rpm until 0.5–1.0 ml of the >30 kD gelatin fraction remained. The gelatin was freeze-dried and then combusted with CuO, and the CO_2_ gas purified. Graphite was prepared by reduction of the sample CO_2_ over an iron catalyst in an excess H_2_ atmosphere prior to AMS radiocarbon measurement [24].

In Groningen (Center for Isotope Research; Lab Code GrA), the bone sample underwent standard chemical cleaning and collagen extraction following Longin [25]. The extracted collagen was combusted into CO_2_ gas which was cryogenically trapped using an automatic device [26], transformed into graphite, and analyzed for ^14^C by AMS [27], [28].

Calibration of individual ^14^C values was performed with the help of Calib 6.0.1 software [29]. However, as it was repeatedly pointed out, the reliable calibration beyond ^14^C age of ca. 19,500 BP remains problematic (e.g. [30]), and at the current stage of research only *estimate* of calendar age can be given.

## Results

The criteria generally used to distinguish cranial material of early dogs from wolves [19] include shortened snout and mandibles (resulting in smaller overall size, crowded teeth and an increased snout width to length ratio) and smaller carnassial teeth (P^4^ and M_1_). However, tooth crowding and snout shortening are also known to occur naturally within some wolf populations and in wolf/dog hybrids [15], [16], suggesting that tooth crowding by itself may not be an especially useful criteria for distinguishing early dogs.

**Figure 2:**
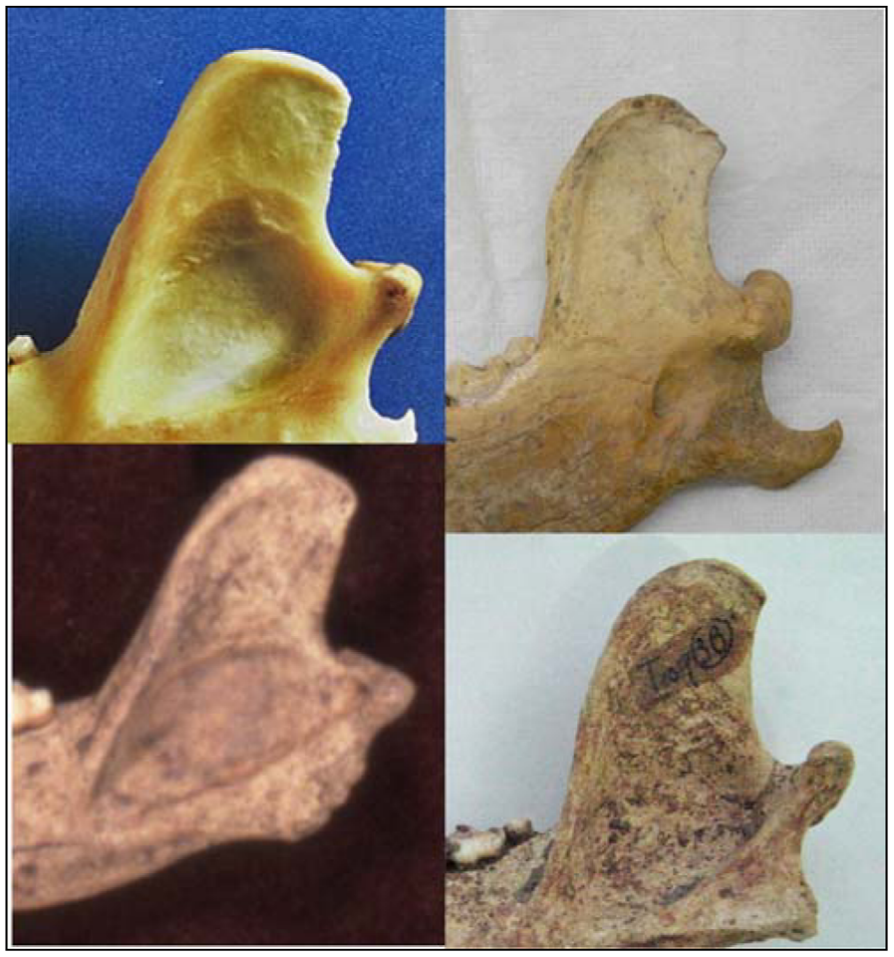
Coronoid process (mandible) profiles, clockwise from bottom left. Thule-age dog (<1000 years old) from Devon Island, central Canadian Arctic [17]; modern Alaskan malamute (Univ. Victoria, Canada 90/28); Razboinichya canid; and Neolithic Chinese dog from Jiahu site [31]. Many Neolithic dogs from the Middle East and North American wolves [32] have a straight profile like Arctic Thule-aged dogs illustrated on the left, while dingo and Chinese wolves [33] have the slightly hooked profile shown on the right. Prehistoric North American dogs outside the Arctic [32], [33] have a profile with a more pronounced hook than the Razboinichya and Jiahu specimens above. Photo credits: Jiahu dog, Yuan Jing; Devon Island dog, Robert W. Park; modern Malamute, Susan J. Crockford; Razboinichya canid, Nikolai D. Ovodov.

The Razboinichya canid cranium is robust with a fairly well-developed stop (Figure 1). Both maxillary and mandibular teeth are compactly arranged. The snout is shortened and relatively broad for its length. The teeth show only slight wear, suggesting this was a relatively young adult animal. The coronoid process of the mandible has the slightly hooked profile seen in Chinese wolves (Figure 2).

The length of the upper carnassial tooth (P^4^) of the Razboinichya canid falls within size the range for modern wolves (Table 1), although the relative length of P^4^ is less than the sum of the two molars (22.9 mm; see Table S2), a value more typical of dogs than wolves [19]. The lower carnassial tooth (M_1_, GL 27.7 mm; see Table S3) falls within the lower range of values for prehistoric wolves and is only slightly smaller than modern European wolves (Table S3). The snout width index is 40.69, which does fit within Clark's [20] Neolithic dogs (range 39.1–45.8) although it is more like the snout breadth ratios given for two putative dogs from the Eliseevichi I site [7], which at 38.6 and 38.7, are only just outside the range these authors give for modern wolves (30.9–36.9) but also outside Clark's [20] Neolithic dog range.

**Table 1:** Razboinichy canid (“Razbo.”) skull measurements (in mm) versus the mean of a sample of late Pleistocene wolves from Předmosti, central Europe [14]; modern wolves from North America [15]; prehistoric Greenland dogs [15]; and two late Glacial canids from Eliseevichi I (“Elis.”), western Russia [7].

Measurement*	Razbo. canid	Předmosti wolves(n = 6–10)**	Modern wolves(n = 66)	Greenland dogs(n = 18)	Elis. canid 5298	Elis. canid 23781
1	211.0	258.6	247.8	206.7	240.0	256.0
2	199.0	n/a***	231.7	194.8	n/a	n/a
3	187.0	227.5	219.2	185.3	213.5	223.0
12	87.2	128.4	110.7	88.7	99.0	100.0
**18 (P^4^)**	**22.6**	**n/a**	**24.6**	**20.3**	**23.7**	**27.2**
30	118.3	140.3	132.8	120.4	145.7	148.0
34	72.0	81.0	77.9	72.0	87.5	91.0

*Numbers are after [13].

**Sample sizes vary among dimensions measured; original means of two sub-samples combined.

***n/a – non-applicable.

The tooth crowding index for the mandible of the Razboinichya canid is 54.94, well below the values for Clark's [20] Neolithic dog sample (range 86.3–103.0) and thus more like modern wolf. However, although Benecke [34] reports slight tooth crowding (index value 99.4) for a small proportion of Předmosti wolf mandibles [34], the index for the “uncrowded” sample is also very high (91.2): both are more like Clark's [20] Neolithic dogs than modern wolves even though the teeth are larger than modern wolves. Such high overall values suggest that the tooth crowding index may be of limited usefulness in distinguishing early dogs unless there is associated corroborative evidence.

**Figure 3:**
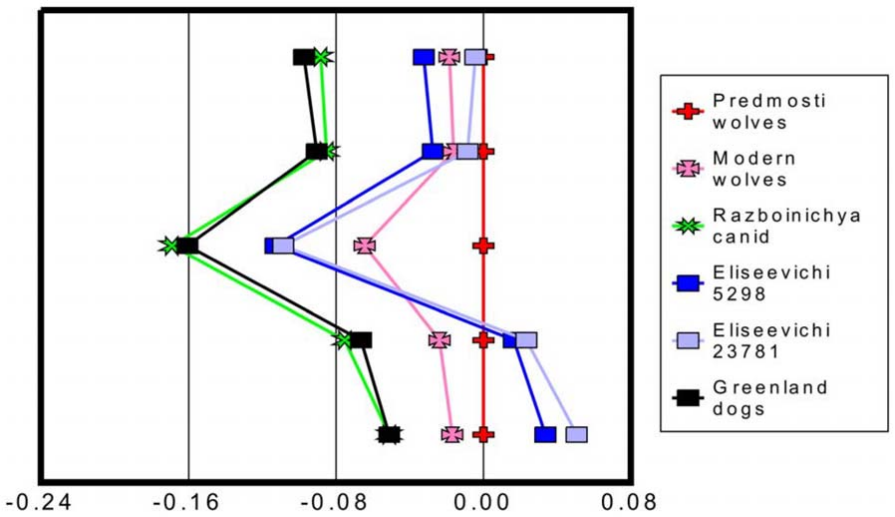
Comparison of some cranial dimensions of the Razboinichya canid (diamonds) and other similar sized canids to a designated “standard” (crosses) comprised of ca. 31,000 cal BP wolves from Předmosti [**14**]. The other canids include modern wolves from North America [15], ancient Greenland Eskimo dogs <1000 years old [17], and two putative dogs from the post-LGM Eliseevichi I site [7]. Negative values are smaller than the ancient wolf standard, positive values are larger. The measurement data are log10 transformed and include (top to bottom): dimension #1 (total length), #3 (basal length), #12 (snout length), #30 (zygomatic breadth), #34 (maximum palate width) [13].

Figure 3 is a simple ratio diagram [21] that compares the basic skull shape of ancient wolves (ca. 31,000 cal BP) from eastern Europe to the Razboinichya canid and other modern and prehistoric wolves and dogs of similar size. Both Eliseevichi canids are more similar to each other than they are to ancient Greenland dogs and both have markedly wider snouts than any other canids. All of these canids, including modern wolves, have markedly shorter snouts than late Pleistocene wolves from Předmosti. The Razboinichya Cave cranium is virtually identical in size and shape to prehistoric Greenland dogs.

Therefore, while the skull falls within the metric criteria of Neolithic and later dogs, the carnassial teeth of the Razboinichya canid are not markedly smaller than those of wolves nor are the tooth rows as distinctly crowded as Neolithic dogs. We conclude, therefore, that this specimen may represent a dog in the very early stages of domestication, i.e. an “incipient” dog, rather than an aberrant wolf. As this canid material pre-dates the LGM and additional putative dogs are not found until thousands of years later, in the late Glacial – early Holocene (ca. 14,000–11,500 cal BP), we conclude that the lineage represented by the incipient dog from Razboinichya Cave did not survive the LGM.

Radiocarbon AMS dating of the Razboinichya canid specimen was performed at three laboratories, Tucson, Oxford, and Groningen (Table 2). The uncalibrated ages are between ca. 27,850 BP and ca. 29,950 BP, thus within ±2 sigma range. The weighted average of the three mandible sample ^14^C dates is 28,790±110 BP, and of four ^14^C values it is 28,800±110 BP. Applying the IntCal09 calibration curve [29], the calendar age estimate is 33,000–33,500 cal BP (at ±2 sigma).

**Table 2:** Radiocarbon AMS dates of the Razboinichya canid.

Sample, year	^14^C date, BP	Calibrated age, cal BP(±2 σ)*	Lab Code	δ^13^C, ‰	δ^15^N, ‰	C/N ratio	Collagen yield, %	C yield, %
Piece of skull (2007)	29,915±1000†	36,490–32,040	AA-76773	−19.3	+8.8	3.2	6.4	35.2
Piece of mandible (2008) (sub-sample 1)	29,650±730†	36,190–32,160	AA-82694	−18.3	+8.5	3.3	4.1	32.0
Piece of mandible (2008) (sub-sample 2)	27,850+150/−140	32,600–31,490	GrA-42167	−19.2	n/a	n/a	2.6	19.0
Piece of mandible (2008) (sub-sample 3)	29,950±170	35,030–34,140	OxA-20923	−18.1	n/a**	3.4	6.0	41.1

† Expressed uncertainty includes both measurement uncertainty and a contamination correction [35].

* Calib Rev. 6.0.1 software [29].

** Single measurement of +8.4‰ was made without standard for nitrogen; therefore, it is considered here.

The specimen is well preserved (Table 2) with collagen yield of 2.6–6.4%; and C/N ratio of 3.2–3.4, well within the accepted limits of 2.9–3.6 [36]. Collagen and carbon yields are very reasonable for specimen older than ca. 20,000 BP, and therefore the ^14^C ages are very reliable in terms of quality of dated collagen. This gives us high degree of confidence that the results obtained are not distorted by any complication factors common when the collagen of old bones is being dated (e.g. [37], [38]). This makes the age determination for the Razboinichya skull and mandibles very secure.

## Discussion

The ^14^C dates for the Razboinichya canid are commensurate with a putative dog skull reported recently from Goyet Cave [6]. Surprisingly, no measurements from this particular specimen are reported. However, one of Germonpré *et al.*'s [6] analyses grouped measurements of the Goyet canid with four other Paleolithic canids, including the two Eliseevichi specimens mentioned above [7]. As a result, Germonpré *et al.* [6] concede that the mean total length of these five putative Paleolithic dog skulls was not significantly smaller than prehistoric wolves and that like the Razboinichya canid, the carnassial teeth were not reduced in size. We conclude, therefore, that the large Goyet canid is also an incipient dog whose lineage did not survive the LGM.

In the vicinity of Razboinichya Cave, several Middle and Upper Paleolithic caves and open air sites exist that have traces of human occupation for the last ca. 50,000–100,000 years or more [39]. These people appear to have been relatively sedentary hunter-gatherers who stayed in one place for many months at a time [40]. Thus, the main occupation of the region took part before the LGM [39], [40]. Reduced human occupation of the Altai region continued through the LGM ([41]; see Figure S4) but apparently, without dogs, perhaps because humans in northern Eurasia became somewhat less sedentary [42]. Not until the Ice Age began to wane did the human settlement patterns conducive to domestication of wolves become common again, i.e. year-round sedentism or sedentary hunter-gathering.

Morphological, behavioral and genetic evidence all suggest that dogs evolved from ancient wolves [1], [2], [8]–[10], probably several times. Traditional anthropological definitions of domestication consider the process to be a deliberate act of selection by humans [1]. However, this view has been challenged in recent years by the hypothesis that animals colonized anthropogenic environments of their own volition and evolved into new (“domestic”) species via natural evolutionary processes because it better fits a number of associated observations, including the evidence from genetics that domestication took place multiple times over geographic space and chronological time in virtually all mammalian taxa [2], [43]. After initial changes occurred, the resulting new species were modified during their association with people via natural adaptation, human selection, and genetic drift.

Since dog domestication almost certainly occurred multiple times without direct human selection, we suggest that it must have occasionally failed. That is, the particular set of ecological conditions associated with human settlement and hunting practices that were necessary to initiate the domestication process must have, on some occasions, existed only long enough to produce a few modified wolves (i.e. incipient dogs) with short-lived lineages.

### Conclusions

We suggest that the pre-LGM Goyet and Razboinichya canids are unlikely to be the ancestors of post-LGM dogs. These canids most probably are both “proto” or incipient dogs that did not persist long enough to found enduring lineages, since no putative dog remains have been found at adjacent sites in western and central Europe and in Siberia occupied during the LGM. The ecological changes caused by progressive cooling almost certainly caused social and settlement pattern changes severe enough to have disrupted the domestication process and prevented the evolution of fully domesticated dogs.

Wolves appear to have been especially attracted to permanent or semi-permanent human settlements. Persistent dog lineages arose in Europe, the Middle East, and China by the end of LGM – early Holocene. By ca. 14,000 cal BP, dogs had become a consistent component of human settlements and were subject to deliberate burial themselves and were included in human graves [1].

Remains of both incipient dogs and early true dogs are critical indicators that a particular set of natural ecological conditions and human-mediated social factors existed at certain times in the past. Mapping the geographic extent and chronological order of these events enriches our understanding of human history and evolutionary processes. The fact that the Razboinichya canid is likely an early incipient dog rather than the oldest ancestor of modern dogs in no way detracts from its historical or biological importance.

## Supporting Information

Figure S1 **Location of the Razboinichya Cave in Altai Mountains, southern Siberia (cave is marked by black triangle).**
(TIF)

Figure S2 **Skull of the Razboinichya canid indicating sampling location for ^14^C AMS dating (black arrow).**
(TIF)

Figure S3 **Mandibles of the Razboinichya canid (A – right; B – left), indicating sampling location for ^14^C AMS dating (black arrows).**
(TIF)

Figure S4 **Frequency of occupation episodes for the Paleolithic sites in the Altai Mountains (after** [**41**] **, with additions). The LGM corresponds to ca. 22,000–16,000 uncalibrated ^14^C years (BP)** [**5**] **.**
(TIF)

Table S1 **Cranium measurements of the Razboinichy canid.**
(DOC)

Table S2 **Mandible measurements for the Razboinichy canid.**
(DOC)

Table S3 **Selected mandible measurements from Razboinichy canid (“Razbo”; this study) versus Pleistocene wolves from Předmosti** [**14**] **, modern wolves** [**16**] **, and prehistoric Greenland dogs** [**15**] **.**
(DOC)
