# Supplementary figures and images for: A 33,000-Year-Old Incipient Dog from the Altai Mountains of Siberia: Evidence of the Earliest Domestication Disrupted by the Last Glacial Maximum
Source: PLoS One. 2011 Jul 28;6(7):e22821. doi: 10.1371/journal.pone.0022821 (PMC3145761; doi:10.1371/journal.pone.0022821)

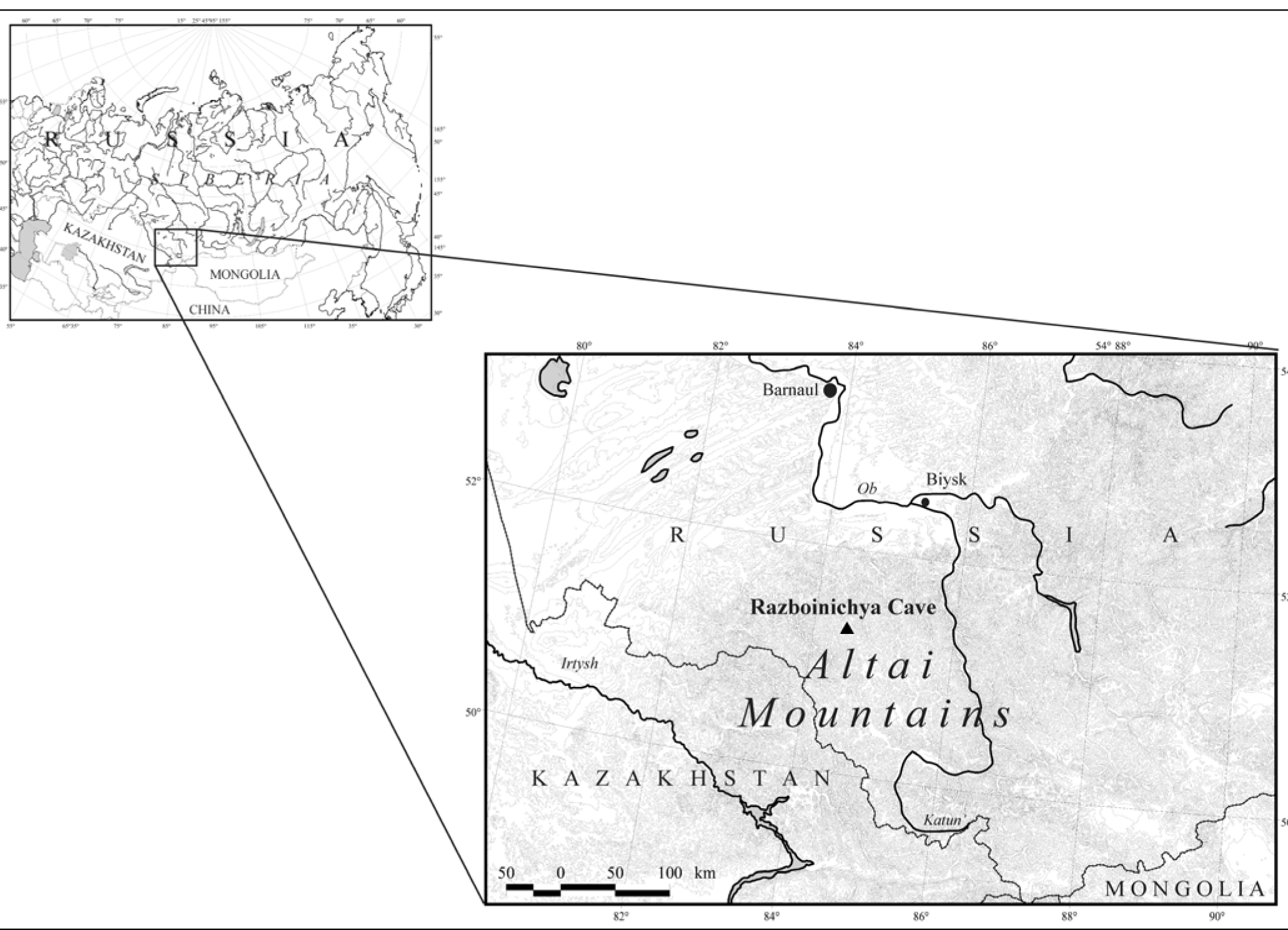

Supplement: Figure S1 — Location of the Razboinichya Cave in Altai Mountains, southern Siberia (cave is marked by black triangle). (TIF) [file pone.0022821.s001.tif]

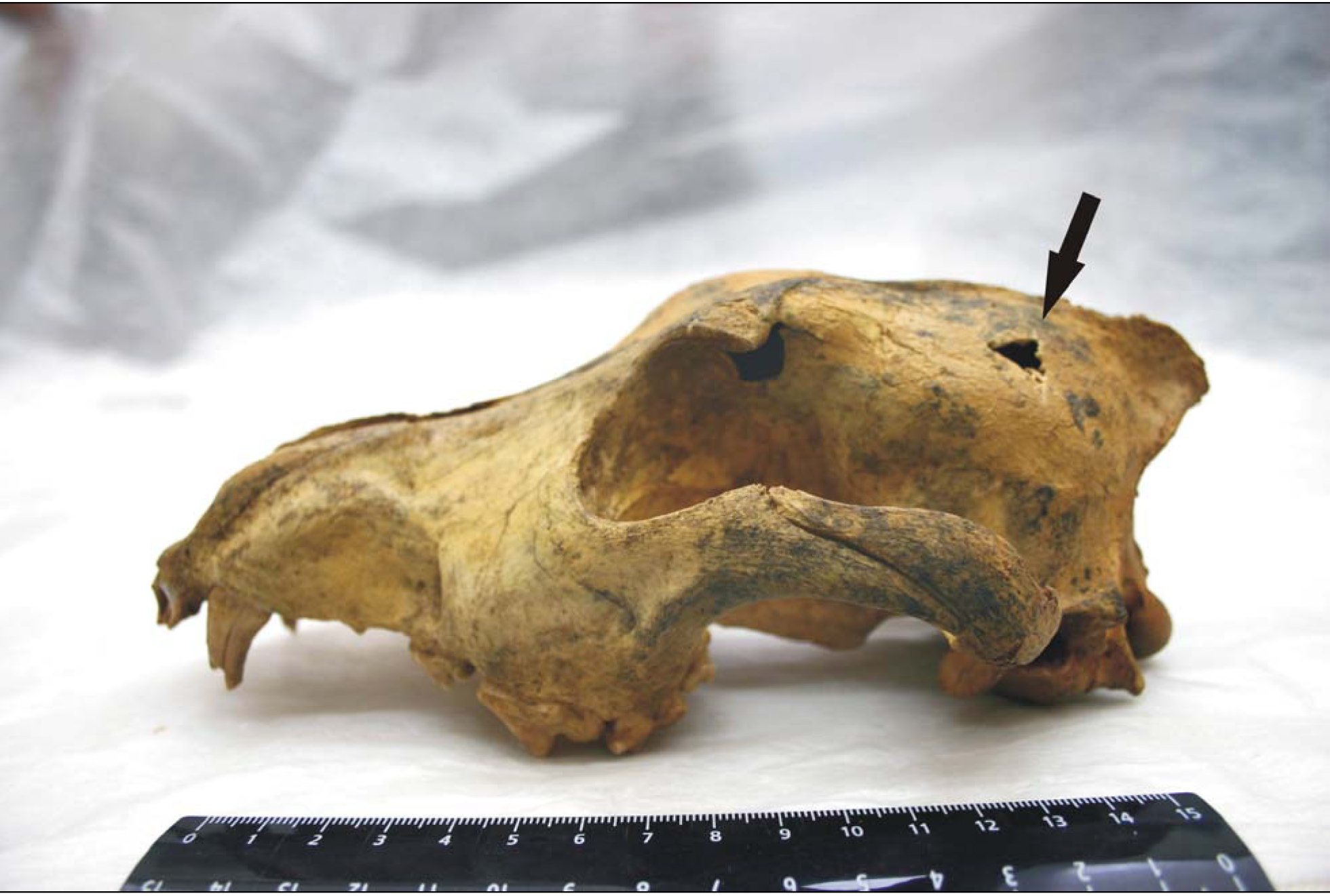

Supplement: Figure S2 — Skull of the Razboinichya canid indicating sampling location for 14C AMS dating (black arrow). (TIF) [file pone.0022821.s002.tif]

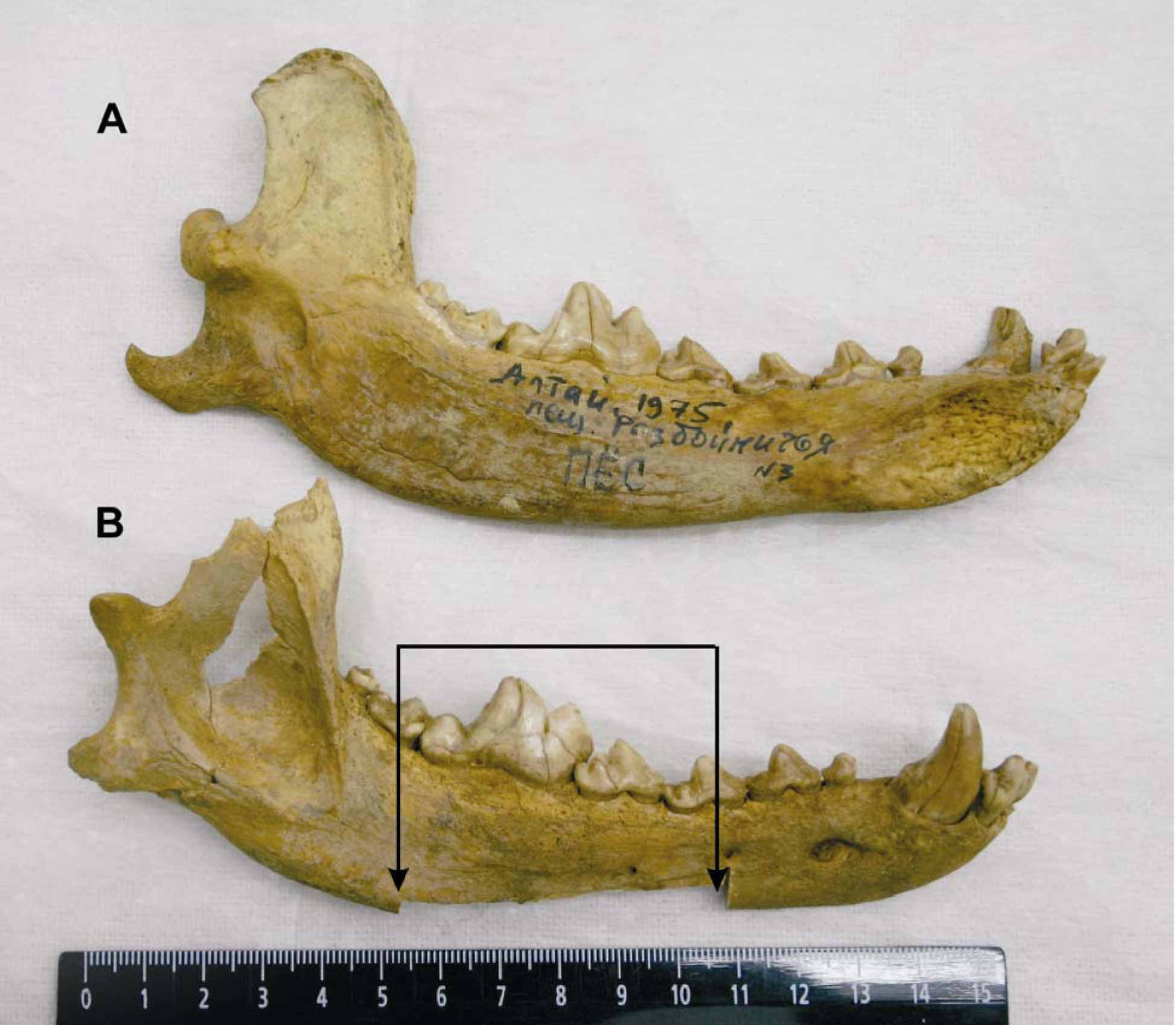

Supplement: Figure S3 — Mandibles of the Razboinichya canid (A – right; B – left), indicating sampling location for 14C AMS dating (black arrows). (TIF) [file pone.0022821.s003.tif]

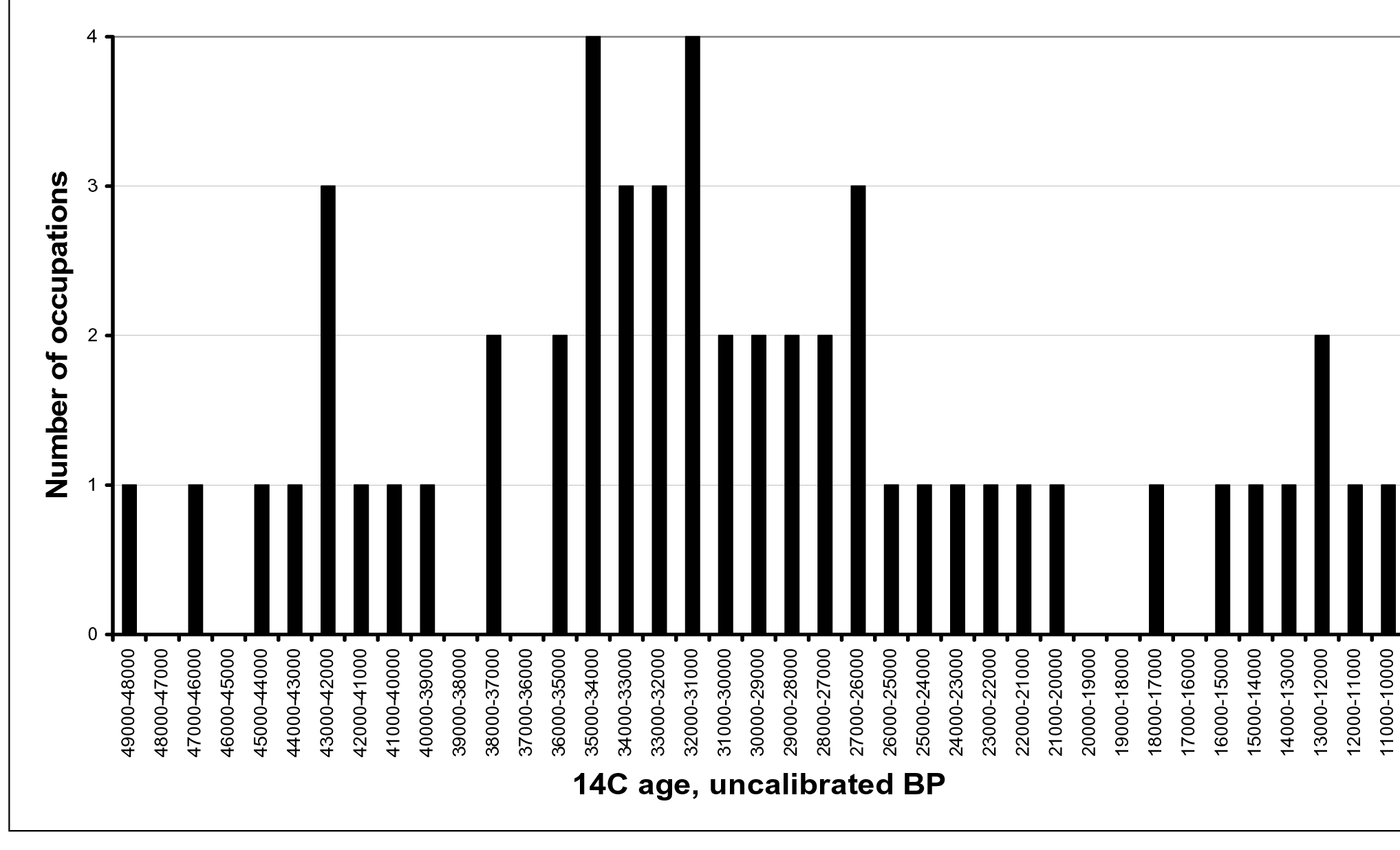

Supplement: Figure S4 — Frequency of occupation episodes for the Paleolithic sites in the Altai Mountains (after [41] , with additions). The LGM corresponds to ca. 22,000–16,000 uncalibrated 14C years (BP) [5] . (TIF) [file pone.0022821.s004.tif]
