# Supplementary material for: A 33,000-Year-Old Incipient Dog from the Altai Mountains of Siberia: Evidence of the Earliest Domestication Disrupted by the Last Glacial Maximum
Source: PLoS One. 2011 Jul 28;6(7):e22821. doi: 10.1371/journal.pone.0022821 (PMC3145761; doi:10.1371/journal.pone.0022821)
Supplement: Table S1 — Cranium measurements of the Razboinichy canid. (DOC) [file pone.0022821.s005.doc]

**Table S1. Cranium measurements of the Razboinichy canid.**

| Dimension #* | (mm) |
| --- | --- |
| 1 | 211 |
| 2 | 199 |
| 3 | 187 |
| 4 | 50.7 |
| 5 | 136 |
| 7 | 100.3 |
| 8 | 103.7 |
| 9 | 119.4 |
| 10 | about 81 |
| 12 | 87.2 |
| 15 | 79.9 |
| 16 | 22.2 |
| 17 | 62.2 |
| 18−20 | − |
| P4/−L | 22.6 |
| P4/−GB | 13 |
| P4/−B | 9.2 |
| M1/−L | 14.9 |
| M1/−B | 17.7 |
| M2/−L | 8 |
| M2/−B | 11 |
| 22 | 24.8 |
| 23 | 72.3 |
| 24 | 69.3 |
| 25 | 46 |
| 26 | 57.2 |
| 27 | 28.1 |
| 28 | 18.4 |
| 29 | 64 |
| 30 | 118.3 |
| 31 | 42 |
| 32 | 58 |
| 33 | 39.9 |
| 34 | 72 |
| 35 | 41.9 |
| 36 | 42.2 |
| 37 | 33.2 |
| 38 | 67.3 |
| 39 | 63.1 |
| 40 | 56 |

*Dimensions are after [15].
