# Supplementary material for: A 33,000-Year-Old Incipient Dog from the Altai Mountains of Siberia: Evidence of the Earliest Domestication Disrupted by the Last Glacial Maximum
Source: PLoS One. 2011 Jul 28;6(7):e22821. doi: 10.1371/journal.pone.0022821 (PMC3145761; doi:10.1371/journal.pone.0022821)
Supplement: Table S2 — Mandible measurements for the Razboinichy canid. (DOC) [file pone.0022821.s006.doc]

**Table S2. Mandible measurements for the Razboinichy canid.**

| Dimension #* | (mm) |
| --- | --- |
| 1 | 153.5 |
| 2 | 160.8 |
| 3 | 149 |
| 4 | 131.4 |
| 5 | 126.2 |
| 7 | 90.8 |
| 8 | 85 |
| 10 | 43.1 |
| 11 | 46.7 |
| 12 | 40.2 |
| 13 | 27.7 |
| 14 | 26.7 |
| 18 | 68 |
| 19 | 27.3 |
| 20 | 21.2 |
| M/1−L | 27.7 |
| M/1−B | 11.1 |
| M/2−L | 10.5 |
| M/2−B | 8.1 |
| M/3−L | 5.6 |
| M/3−B | 5.7 |

*Dimensions are after [15].
