# Supplementary material for: A 33,000-Year-Old Incipient Dog from the Altai Mountains of Siberia: Evidence of the Earliest Domestication Disrupted by the Last Glacial Maximum
Source: PLoS One. 2011 Jul 28;6(7):e22821. doi: 10.1371/journal.pone.0022821 (PMC3145761; doi:10.1371/journal.pone.0022821)
Supplement: Table S3 — Selected mandible measurements from Razboinichy canid (“Razbo”; this study) versus Pleistocene wolves from Předmosti [14] , modern wolves [16] , and prehistoric Greenland dogs [15] . (DOC) [file pone.0022821.s007.doc]

**Table 3. Selected mandible measurements from Razboinichy canid (“Razbo”; this study) versus Pleistocene wolves from Předmosti [8], modern wolves [16], and prehistoric Greenland dogs [9].**

| Dimension #* | Razbo | Předmosti | | | Modern wolves | | | Greenland dogs | |
| --- | --- | --- | --- | --- | --- | --- | --- | --- | --- |
|  |  | mean | n | range | mean | n | range | mean | n |
| 1 | 153.5 | 185.7 | 25 | 162.0–202.0 | − |  | − | 149.0 | 17 |
| 8 | 85.0 | 97.9 | 53 | 90.7–106.5 | − |  | − | − |  |
| **13 (M_1_)** | **27.7** | **30.0** | 91 | **27.5–32.5** | **28.96** | 6 | **27.9–31.9** | **23.5** | 14 |

*Dimensions are after [15].
